# Antioxidant and Cytoprotective Effect of Quinoa (*Chenopodium quinoa Willd*.) with Pressurized Hot Water Extraction (PHWE)

**DOI:** 10.3390/antiox9111110

**Published:** 2020-11-11

**Authors:** Eng Shi Ong, Charlene Jia Ning Pek, Joseph Choon Wee Tan, Chen Huei Leo

**Affiliations:** Science, Math & Technology, Singapore University of Technology & Design, Singapore 487372, Singapore; cpek002@e.ntu.edu.sg (C.J.N.P.); joseph_tan@sutd.edu.sg (J.C.W.T.); chenhuei_leo@sutd.edu.sg (C.H.L.)

**Keywords:** Quinoa, pressurized hot water extraction (PHWE), LC/UV, LC/MS, principal component analysis (PCA), antioxidant properties, cytoprotective effect

## Abstract

Quinoa is widely noted for its nutritional value. The seed is the main edible part of the plant and exists in at least three different colors: white, red and black. This study utilized a pressurized hot water extraction (PHWE) for the extraction of phytochemicals from quinoa. Chemical fingerprints with LC/UV and LC/MS using a targeted approach and pattern recognition tools were used to evaluate the quinoa extracts. The antioxidant properties for various types of quinoa were evaluated using DPPH assay, ABTS assay and the cytoprotective effect of quinoa extracts were investigated in HMEC-1 cell line. Distinctive chemical profiles obtained from black and red quinoa were well correlated with the antioxidant activities and cytoprotective effects. The combination of PHWE, chemical standardization with LC/UV and LC/MS, pattern recognition tools and biological assay provided an approach for the evaluation and eventual production of quinoa extracts for functional food.

## 1. Introduction

Quinoa (*Chenopodium quinoa Willd*.) is characterized as a broad leaf plant (non-grasses) and its seeds have been incorporated into regular cereal-based foods. It is a dicotyledonous plant that belongs to the Chenopodiaceae family and is one of the oldest native crops originated from the Andean region in South America [1]. Quinoa seeds are the main edible parts of the plant and exist in at least three varieties, with different colors such as white, red and black. In recent years, quinoa has received much attention due to its exceptional high nutritional value and potential health benefits. Specifically, quinoa seeds are gluten-free, have a low glycaemic index, and contain an excellent balance of essential amino acids, fiber, lipids, carbohydrates, vitamins, and minerals [2].

Quinoa or quinoa products are rich in not only macronutrients such as protein, polysaccharide and fats, but also consist of a number of bioactive phytochemicals such as flavonoids, carotenoids, fiber, saponins, vitamins, fatty acids and others [3,4]. In addition, saponins are reported to contribute to the bitter taste of quinoa and are an important source of anti-nutrients, which may have negative effects on the growth of monogastric animals or humans that relies on quinoa as their primary source of food energy [1,2,3,4,5]. The presence of these phytochemicals may contribute to diverse beneficial physiological properties such as antioxidant activity [2,5], leading to protection against a number of oxidative stress-related diseases, such as diabetes and reproductive diseases [6,7,8,9,10,11,12,13]. However, the composition and bioactivity of these beneficial phytochemicals are different between the different colored quinoa seeds. Specifically, darker colored quinoa have higher levels of phenolic compounds, contributing to greater antioxidant capacity [4].

Despite their high levels of phytochemicals and promising beneficial effects as a food product, most studies on quinoa extracts have used organic solvents such as methanol and ethanol, which are commonly used to extract the constituents present in the botanicals [14,15,16]. Extraction method utilizing organic solvents would require proper waste disposal and solvent removal from the end product before they are ready for consumption. Hence, organic solvent extraction methods are not environmentally friendly and sustainable. Thus, selection of the green extraction technique is an important step that can greatly affect the end product [17,18]. Indeed, modern extraction techniques such as pressurized hot water extraction (PHWE) has become more widely accepted for the extraction of bioactive compounds from a wide variety of plant and food sources [17,18,19]. At the same time, PHWE was used for the sequential extraction of saponins, xylan and cellulose from quinoa stalks [20]. In PHWE, only water is used and temperature may be adjusted to optimise the extraction efficiency and selectivity [21]. Chemical fingerprint with HPLC, HPLC/MS and other analytical techniques with pattern recognition tools such as principal component analysis (PCA) and others are commonly used for the evaluation of botanical extract. To get to a set of components that is biased towards describing variation related to a particular feature such as class, supervised methods of analysis such as orthogonal projection to latent structure discriminant analysis (O-PLS-DA) are used [22,23,24].

The aim of this study is to utilize a PHWE method for the extraction of bioactive phytochemicals from different types of colored quinoa. For the current work, LC/MS analysis and antioxidant assay (in vitro) will be used to evaluate the properties of the extracts obtained. Specifically, the optimum PHWE extraction efficiency will be evaluated with normalization to constant sum, chemical fingerprint, pattern recognition tool and DPPH assay. Finally, the biological activities of the different colored quinoa will be further characterized using cytoprotective assays (in vitro).

## 2. Materials and Methods

### 2.1. Chemicals and Quinoa Seeds Materials

Black, red, Bolivian- and Peru-grown white quinoa seeds were purchased from the supermarkets. 1,1-diphenyl-2-picrylhydrazyl (DPPH), ascorbic acid (ASC), HPLC grade water and methanol, seasand, formic acid, were purchased from Sigma-Aldrich (Singapore). Cell culture reagents, trypsin and Fetal Bovine Serum (FBS), Attachment Factor (AF), 0.4% trypan blue, l-glutamine acid, MCDB-131 and Dulbecco’s Modified Eagle Media (DMEM) were purchased from Thermofisher Scientific (Singapore). All other chemicals and solvents that were used throughout the experiments were standard analytical grade.

The quinoa samples were dried at 40 °C in a laboratory oven for 24 h. The samples were crushed using mortar and pestle after the samples cooled down, before sieving could be carried out. Samples were sieved individually to separate into the following particle sizes: below 0.3 mm, between 0.3 and 2.8 mm, and above 2.8 mm. Chemical analysis was performed on fine samples with particle sizes below 0.3 mm. The reduced particle size generates a more homogenous and representative sample.

### 2.2. XRF Determination of Quinoa Seeds

For X-ray screening analysis of the samples, a Bruker TRACER 5 i Portable X-Ray Fluorescence Analyzer (Berlin, Germany) was used (pXRF). The pXRF is equipped with Rhodium thin window X-ray tube and silicon drift detector with <140 eV @ 250,000 cps Mn Kα. The output energy spectrum produced is analyzed using the software-Artax 8.0.0.443 (Berlin, Germany). A filter (Cu 75 µm, Ti 25 µm, Al 200 µm) was applied in the pXRF analyzer to enhance the detection of heavy metals like Hg, As, Br and Pb in the energy spectrum produced. The filter is selected to accentuate the peaks of the heavy metals that are causes of concern for toxicity from the sample. The background radiation scatter and any excitation due to lower energy peaks is eliminated. Thus, use of the filter could better identify the presence of these metals of interest.

XRF cups, which are double open-ended cups, were used to hold the samples. A 4 µm thick prolene thin sheet was fastened to one side of the XRF cup with a ring to create a taut prolene-film window. A sample of 1.0 g (particle size < 0.3 µm) was added through the open side of the cup and weighed to a determined weight. Another film was used to close the open side of the XRF cup. The sample cup was placed on the window of the pXRF analyzer. The tube voltage was set at 50 kV, current at 35 µA and with an exposure or run time of 300 s, with three readings taken each time for each sample.

After the elemental spectrum was obtained through Artax, elements were identified from the peaks and deconvolution process was carried out. A total of 45 elements was commonly found across all samples. Hence, the elements were saved into the method file that was used to identify elements in subsequent XRF runs.

### 2.3. PHWE System

For the extraction of target compounds from each sample, a laboratory system shown in Figure S1 was assembled similar to our earlier report [18]. The system included a stainless steel extraction cell (250 × 10 mm i.d.), an isocratic Shimadzu LC10 series pump (Kyoto, Japan), and a constant temperature oven (Hewlett Packard 5890 Series II). To ensure efficient heat transfer, stainless steel tubings 1/16 in. o.d. and 0.18 mm i.d. were used for all connections. An accurately weighed 0.5 g sample was placed into 20 mL scintillation vials. The sample was mixed with a small proportion of sand before loading into the extraction cell. The pump was set to a constant flow rate of 1.2 mL/min for 40–45 min. Back pressure was maintained by the presence of sand. The pressure recorded was 10–15 bar, as indicated by the pump. Oven temperature was varied from 80 to 100 °C. The extract obtained was top up to a 50 mL volumetric flask. The extracts obtained from 0.5 g of original samples to 50 mL of water were diluted for subsequent DPPH and cytoprotective assay.

### 2.4. LC/UV/MS Profiling of Quinoa Seed

LC/UV/MS was used to determine the phytochemical characteristic profiles in different quinoa extracts. A C18 reverse phase HPLC column (Zorbax SB-C18- 3.5 microns, 4.6 × 100 mm, Agilent, Santa Clara, CA, USA) was used for compound separation. The total flow rate was set at 0.8 mL/min, while the column temperature was maintained at 40 °C. The injection volume was loaded into the system at 10 µL and concurrently separated into both HPLC (Shimadzu Nexera X-2, Kyoto, Japan) and LC-MS (Shimadzu LCMS-8050, Kyoto, Japan) machine. The gradient elution involved a mobile phase consisting of (A) 0.1% of formic acid in water and (B) 0.1% of formic acid in acetonitrile with the gradient parameter performed in a negative mode ([M^−^H^−^]) at 0.10 min 10% of B, 15 min 100% B, 15.10 min 10% B and 25.10 min 10% of B, which was used to re-equilibrate the column to initial conditions. The MS Electrospray Ionisation (ESI) was operated with a nebulizing gas flow of 2.8 L/min, interface temperature of 300 °C, with a heating and dry gas flow of 9 L/min. On the other hand, the UV detector was adjusted to detect bioactive compounds at the absorbance value of 280 nm. The derived extracts were filtered through a 0.2 um filter into vials and placed on the auto-sampling rack for compound analysis. The compounds listed in Table 1 were identified based on various reports and published MSMS spectra [3,4,25,26,27]. In addition, comparison of MSMS spectra at the human metabolome database (HMDB) was done with the various compounds reported (https://hmdb.ca/)

For the purpose of normalization to constant sum, the measured peak intensity of each sample was normalized prior to retrieving the average of the three sample readings. For LC/UV, all peaks at 280 nm were obtained. For LC/MS, the peak intensities that corresponded to the molecular weight (*m*/*z*) in Table 1 were normalized within each sample to the total peak intensity of the sample. Normalization was performed to address the differences in concentration. Additionally, normalized data for each sample were analysed using the Principal Component Analysis (PCA) scores plot for 15 peaks. For PCA, the data were condensed to two principal components to describe the maximum variation between datapoints. From the PCA scores plot, observations can be made based on the clusters, trends and outliers.

### 2.5. Determination of DPPH Radical Scavenging Activities

The antioxidant activity of the quinoa extracts was determined based on the ability to scavenge free radicals using the DPPH (1,1-diphenyl-2-picrylhydrazyl) method. In the first set of experiments, pulverised and non-pulverised white Bolivian quinoa seeds was extracted at different temperatures (80, 100 and 120 °C) to determine the effect of temperature and pulverization of quinoa extract on antioxidant capacity. Briefly, DPPH is light-sensitive and rapidly decolorizes when exposed to light, thus aluminum foil was utilized to shield the prepared volumetric flasks. The blank (200 µL of methanol), control (150 µL of water) and pulverised and non-pulverised white Bolivian quinoa extracts (150 µL) from different temperatures (80, 100 and 120 °C) were transferred into the 96-well plate. Consequently, excluding the blank, 50 µL of DPPH solution were added into each well at the final concentration of 100 µM before wrapping the 96-well plate in aluminum foil for incubation at 37 °C for 30 min. All experiments were performed in triplicates. The degree of absorbance was recorded using a spectrophotometric plate reader (Thermofisher Multiskan GO) at 517 nm.

% inhibition = [(A_0_−A_1_)/A_0_ × 100], where A_0_ refers to the absorbance value of control and A_1,_ the absorbance measured from the extract sample.

In the subsequent set of experiments, the antioxidant capacities of the different colored quinoa extracts (black, white and red) were compared. Similarly, the blank, control and various concentrations of the different colored quinoa extracts (0.34 to 2.5 mg/mL) were prepared into the 96-well plate. Subsequently, excluding the blank, DPPH solution was added into each well (final volume, 200 µL) at the final concentration of 100 µM before wrapping the 96-well plate in aluminum foil for incubation at 37 °C for 30 min. All experiments were performed in triplicates. Ascorbic acid (10 mM) was used as a positive control. The absorbance was recorded using a spectrophotometric plate reader (Thermofisher Multiskan GO) at 517 nm.

% maximum inhibition = [(A_0_−A_1_)/(A_0_−A_p_) × 100], where A_0_ refers to the absorbance value of control, A_1_ refers to the absorbance measured from the extract sample and A_p_ refers to the absorbance measured from Ascorbic acid (10 mM). Concentration–response curves for DPPH inhibition were computer-fitted to a sigmoidal curve using nonlinear regression (Prism version 5.0, GraphPad Software, San Diego, CA, USA) to calculate the sensitivity of DPPH inhibition for quinoa extract (IC50) [28,29,30]. Group IC50 values were compared using one-way ANOVA with post-hoc analysis using Tukey’s test [31,32]. Post-hoc analysis was only performed when the F value was significant and there was no variance in homogeneity [33]. *p* < 0.05 was considered statistically significant.

### 2.6. ABTS Antioxidant Capacity Assay

Stock solutions of 2,2′-Azinobis-3-ethylbenzothiazoline-6-sulfonic acid (ABTS, 7 mM) and potassium persulfate (140 mM) were prepared. Potassium persulfate stock solution (88 µL) was added to ABTS stock solution (5 mL). The mixture was stored in the dark for 16 h. This produced a solution of ABTS^•+^ which was dark purple in colour. The solution was diluted 50-fold in distilled water to give an absorbance of approximately 0.80–0.70 at 734 nm. Several dilutions (100 μM to 1 mM) of vitamin C were prepared for the generation of the standard curve, which will produce between 5% and 50% inhibition of the blank absorbance (ABTS^•+^ alone). Different coloured quinoa (1 mg/mL) were also prepared. Three replicates of each sample were measured by adding 5 μL of each sample to 96-well microplate containing 200 μL of ABTS^•+^ solution. Microplates were incubated at room temperature for 30 min, and absorbances were read at 734 nm using Multiskan GO microplate reader (Thermo Scientific, Vantaa, Finland). The absorbance at 734 nm was calculated as percentage of the absorbance of the uninhibited radical cation solution (Blank) according to the equation

Inhibition of absorbance at 734 nm (%) = (1 − (Af/A0)) × 100

where A0 is the absorbance of uninhibited radical cation (ABTS^•+^ alone) and Af is the absorbance measured 30 min after the addition of antioxidant test samples.

The inhibition of absorbance at 734 nm of standards was plotted as function of their final concentrations. The Vitamin C equivalent antioxidant capacity (CEAC) value of the quinoa samples was calculated using the equation obtained from the linear regression of the standard curve substituted of absorbance at 734 nm values for the quinoa sample. Group mean CEAC values were compared using one-way ANOVA with post-hoc analysis using Tukey’s test as described above.

### 2.7. Cell-Culture and Cytoprotective Effects

Dermal microvascular endothelium (HMEC-1) cells derived from newborn males were purchased from ATCC. This cell line was cultured in a specially prepared medium comprised of basal medium (MCDB-131), 2% FBS, 10–20 ng/mL of Recombinant Human Epidermal Endothelial Growth Factor (EGF), 10 µm of L-glutamic acid and maintained at the incubation temperature of 37 °C at 5% CO_2_. The IC50 values determined from the DPPH assay were used for the cytoprotective assay.

To evaluate the possibility of cytoprotective effects exerted by the quinoa extracts, cell counting was implemented. In preparation for this assay, approximately 30,000 cells were seeded into the 6-well plate and left to adhere and proliferate for 24 h. Following this, the H_2_O_2_ concentration of 20 mM was further diluted to 0.1 mM. The cells were treated with 0.1 mM H_2_O_2_ over a period of 48 h in 37 °C, 5% CO_2_ incubator with a fresh change of treatment medium after an initial 24 h to induce damage to the HMEC-1 cells. Cells that were not treated with H_2_O_2_ served as control. To determine if the different colored quinoa extracts possess the ability to prevent H_2_O_2_-induced cell death, 0.001 g/mL of filtered quinoa extract was co-administered to the cells. All cell treatments are performed in duplicate. The cells were monitored and observed for morphological alterations over the 48 h period. After 48 h of treatment, the cells were trypsinised and cell viability of each well was determined by cell counting. Group mean cell count values were compared using one-way ANOVA with post-hoc analysis using Tukey’s test as described above.

## 3. Results and Discussion

### 3.1. Characterization of Minerals Present in Quinoa Seeds by XRF

**Figure 1:**
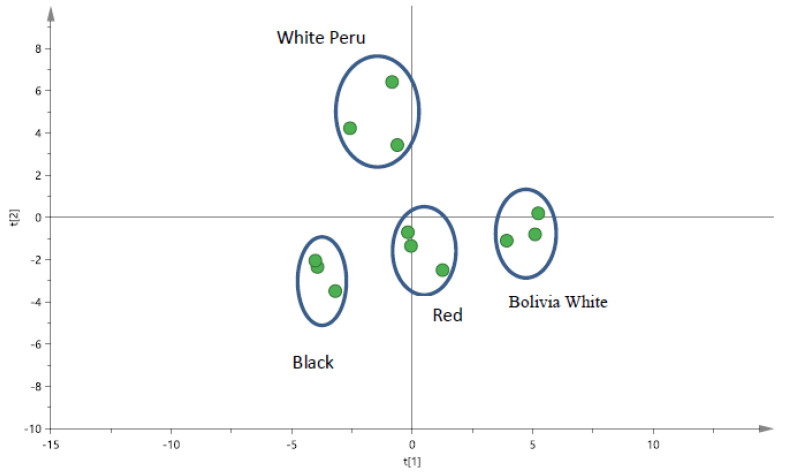
PCA score plot for quinoa seeds samples (pulverised) by pXRF for black quinoa seeds, red quinoa seeds and two different types of white quinoa seeds (Bolivia and Peru).

XRF spectroscopy is an excellent technique for qualitative and quantitative analysis of the material composition at the external part of a solid sample. For the current work, the minerals present in various types of quinoa seeds were determined by XRF. From the PCA score plot in Figure 1, it was noted that varying amount of minerals such as K, Ca, P, Fe, Cu and Zn, respectively, were found in different types of quinoa seed. The grinding and sieving of the seed samples ensured that a more homogeneous sample can be obtained. In addition, it will allow the investigation of components that were present at the outer and possibly the inner core of the seeds. From Figure S2, it was noted that minerals such as Ca and K were found to be significantly higher (*p* < 0.05) in the non-pulverized (whole seed) as compared to the pulverized seeds in the different-coloured quinoa. In contrast, other minerals such as Fe, Cu and Zn were significantly higher (*p* < 0.05) in pulverized samples when compared to the whole seed for most cases, with the exception of Peru white quinoa (Figure S2). Two elements, Ca and K, were found to be present in relatively higher amounts in comparison to other elements. Interestingly, both the black quinoa and Peru white quinoa have significantly higher amounts of Ca in the seeds when compared to the red quinoa and Bolivia white quinoa. Upon pulverization, the amount of Ca were normalized between the different varieties of quinoa but not Peru white quinoa, which was significantly lower (Figure S3). On the other hand, the red quinoa has the highest amount (*p* < 0.05) of Ca present in the seeds compared to other varieties of quinoa seed. This observation remains when the seeds are pulverized (Figure S3). Hence, it was proposed that minerals such as Ca and K may be present at different amounts within the outer core of the seeds. Other elements such as P, Fe, Cu and Zn were proposed to be more evenly distributed in the quinoa seeds.

### 3.2. Optimization of PHWE for Pulverized and Non-Pulverized Seeds

The pulverized and non-pulverized seeds were extracted with PHWE at 80, 100 and 120 °C, respectively. The compounds in quinoa were determined by LC/UV and LC/MS. With reference to Tables S1 and S2, a higher yield of phenolic compounds in pulverised than non-pulverised seeds was observed. From Table S1, it was noted that compounds such as genistein and others portrayed an increased yield based on different extraction temperatures from 80 to 120 °C. For the current work, calibration by external standard was not used, as it was noted that normalization to constant provided an alternative approach for the comparison of target compounds in complex matrix [34,35].

**Figure 2:**
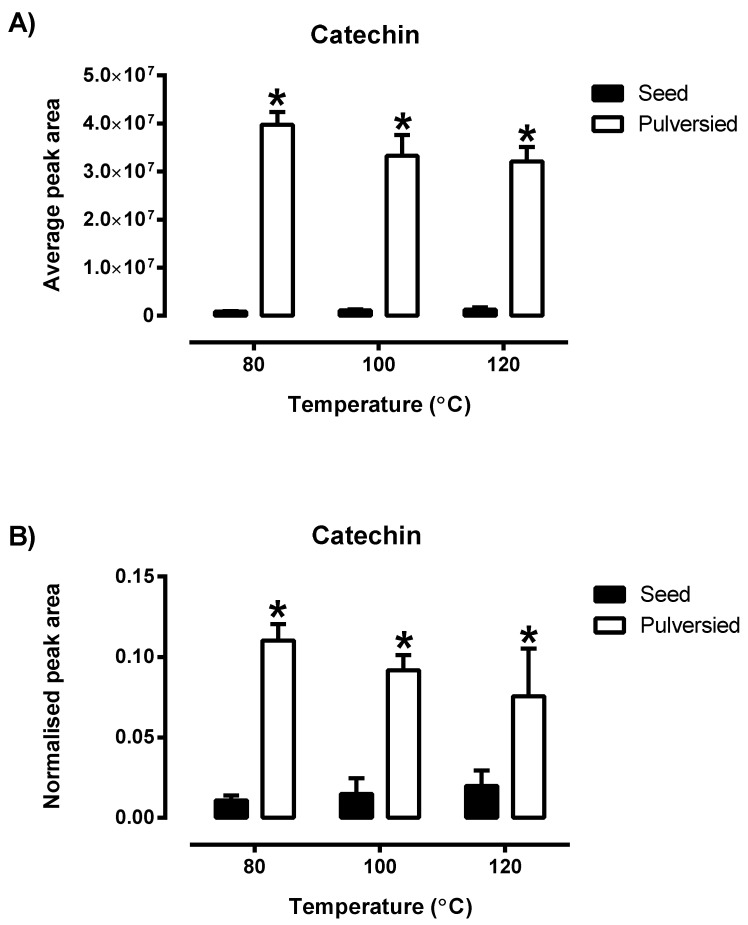
The comparison in the average peak area (**A**) of the phytochemical catechin with the normalised data of the peak area (**B**) derived from Tables S1 and S2 of the pulverised and non-pulverised Bolivian seeds (*n* = 3). Each value is represented by mean ± SD with significance accepted at *p* ≤ 0.05. * Significantly different amount of catechin was found in the pulverised as compared to non-pulverised Bolivia white quinoa (Students’ unpaired *t*-test).

From Figure 2, catechin extracted from the pulverised quinoa was observed to be significantly higher (*p* < 0.05) than the seed throughout the varying extraction parameters. Although the amount of catechin was found to decrease with increasing temperature (Tables S1 and S2), there is no statistical difference in catechin levels between the different extraction temperature. Vanillin is another bioactive component present; a decreasing trend was portrayed in the pulverised seeds while the opposite trend was demonstrated in the non-pulverised samples. Results from both sides were observed to be consistent with the normalised values. It was noted that the amount of certain target compounds such as catechin, vanillin and others was found to decrease when the extraction temperature was increased from 80 to 120 °C. Other marker compounds such as caffeic acid, ferulic acid and others were observed to have an opposing trend. The main reason for the differences in the amount of phytochemical content obtained could have been due to variation in temperature that may result in the degradation of the target compounds.

**Figure 3:**
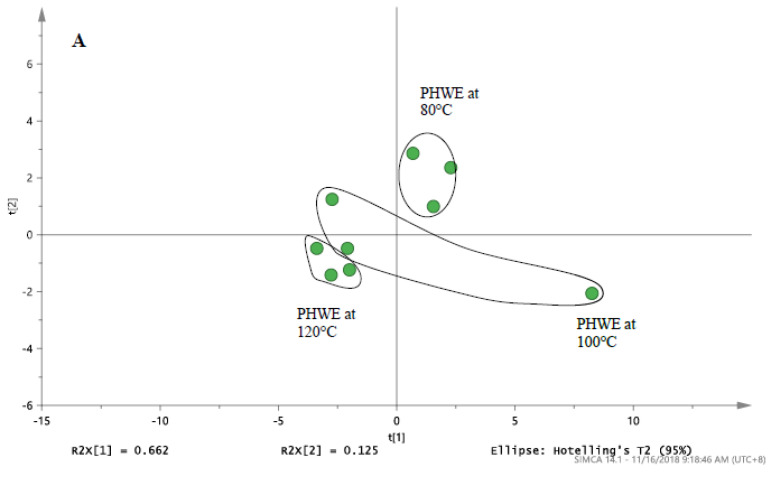

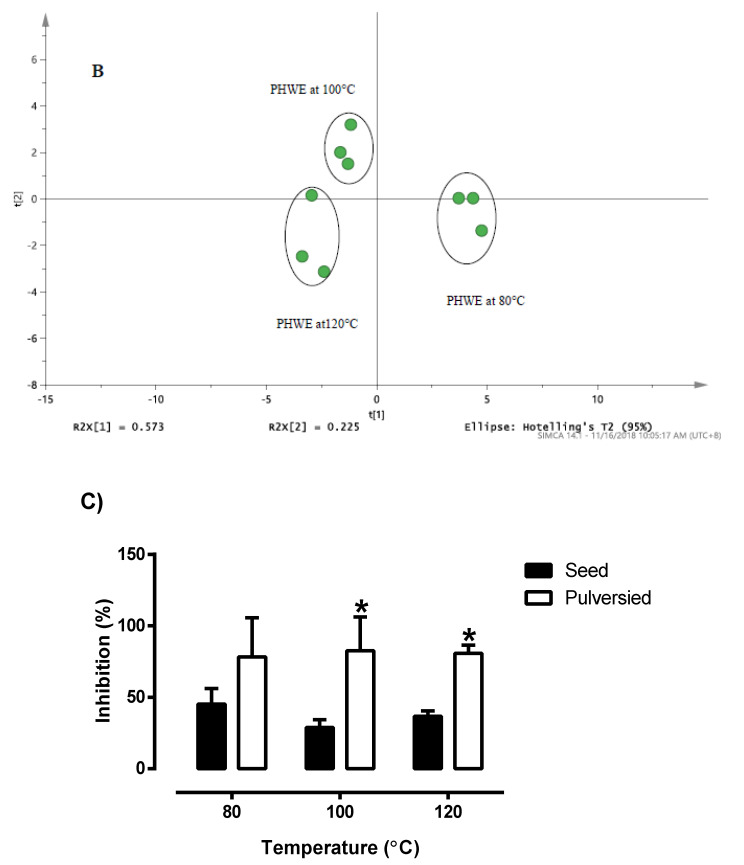
The PCA score plot of (**A**) LC/MS profile for white Bolivia seed derived from Table 1. (**B**) LC/UV profile for white Bolivian quinoa seed detected at 280 nm. (**C**) Radical scavenging activity of pulverised and non-pulverised white Bolivian quinoa seeds extracted at 80, 100 and 120 °C. * Significant difference between the whole seeds and pulverized seed at a given temperature (Students’ unpaired *t*-test). Results are mean ± SD (*n* = 3) with the significance accepted at *p* ≤ 0.05.

Based on the chromatographic profiles obtained at different extraction temperature, the normalized data for selected peaks of each sample were analysed using PCA. PCA was used to differentiate and classify the samples based on the entire chromatograms from LC/UV and targeted ions from LC/MS. For the chemical fingerprint obtained from LC/MS in Table 1 and the PCA score plot in Figure 3A, it was noted that distinctive profiles were obtained from Bolivian seeds extracted at 100 and 120 °C. However, to establish that both temperatures produced similar phytochemical compound would be inconclusive, as the distance between the points still holds a certain gap. While the two may seem synonymous, seeds extracted at 80 °C were observed to have a completely distinct fingerprint from the latter. In conjunction, the PCA score plot from the chromatograms obtained from LC/UV (Figure 3B) illustrated a complementary formation which portrays a degree of similarity between the characteristic profile of quinoa seeds extracted at 100 and 120 °C, while extracts of 80 °C were quite distinct. From this set of data, the variation in the profile of these seeds is most likely due to the different extraction parameters that resulted in the production of a varying amount of phytochemical content.

The holistic nature of botanical extracts creates a challenge in establishing the quality of the product. Based on the monitoring of a single compound, it was challenging to select the optimum temperature of extraction. For botanical drugs, the characteristic profile of chemical constituents can be determined based on chromatographic methods and others (Botanical drug development, guidance for industry, USFDA, 2016). Hence, chemical fingerprinting provided an approach for the quality assessment of the extracts obtained. With regards to the comparison of pulverised and non-pulverised quinoa in Figure 3C, it was evident that the pulverized quinoa seeds had significantly higher (*p* < 0.05) inhibition for DPPH when compared to non-pulverized quinoa seeds. This increment effect was consistent across the different extraction temperature, even though it failed to reach statistical significance (*p* = 0.13) at 80 °C. Furthermore, it was also noted that there is no statistical difference in DPPH inhibition between the various extraction temperatures within the of pulverised and non-pulverised quinoa (Figure 3C). Based on the chromatographic analysis and DPPH assay, it was noted that a higher surface area in pulverised quinoa seeds will ensure that a higher amount of target compounds is extracted. Hence, DPPH assay provided a useful approach for the monitoring of extraction efficiencies at different applied temperatures.

For PHWE, it was proposed that the first step is the adsorption of solutes from the various active sites in the quinoa seed (pulverised and non-pulverised) at the preset temperature and flowrate. The next step will likely involve the diffusion of extraction fluid into the sample. Lastly, the target compounds may partition themselves from the sample into the extraction fluid. Compared to the minerals present (Figure 1), the bioactive compounds were proposed to be present in the inner cores of the quinoa seeds (Figure 3C). Grinding and sieving the seed samples provided a higher exposed surface area. On the whole, apart from the extraction of contents being significantly higher in the pulverised than the latter, the extraction temperature of 100 °C was selected for further experiments.

Based on the various compounds detected, a comparative investigation revealed that similar bioactive components in both Tables S1 and S2 were consistent with the previous study [5]. From our study and other works, it was suggested that the phenolic compounds present in quinoa were proposed to act synergistically. Hence, the monitoring of various bioactive compounds, chromatographic fingerprint and pattern recognition tools provided an approach to establish the quality of quinoa seed extracts. Indeed, flavonoids and their family of polyphenolic compounds are well-recognized for their antioxidant and anti-inflammatory bioactivities [36] which contributes to the improvement in vascular function in diabetes [37,38].

### 3.3. Analysis of Black, Red and White Quinoa by LC/UV/MS

After establishing the temperature of extraction for the phytochemical compounds present in pulverised seeds, extraction of the other pulverised quinoa variety was conducted at 100 °C. Based on the results in Table 1, differences in extraction content between various varieties were observed. It was noted that the p-coumaric acid present in different types of quinoa was found in varying concentrations, the highest being black quinoa, followed by white Bolivian, and red and white Peruvian quinoa. It was observed that white Peruvian quinoa can possess certain compounds that exceed the concentration of other varieties. An example would be the vanillin, where the white Peruvian quinoa has the highest peak area, followed by white Bolivian, red and black (Table 1). The LC/MS and LC/UV chromatograms of the pulverised quinoas can be found in Figure S4, respectively. Thus, though the seeds fall under the quinoa species, their phenolic content remains vastly different.

**Figure 4:**
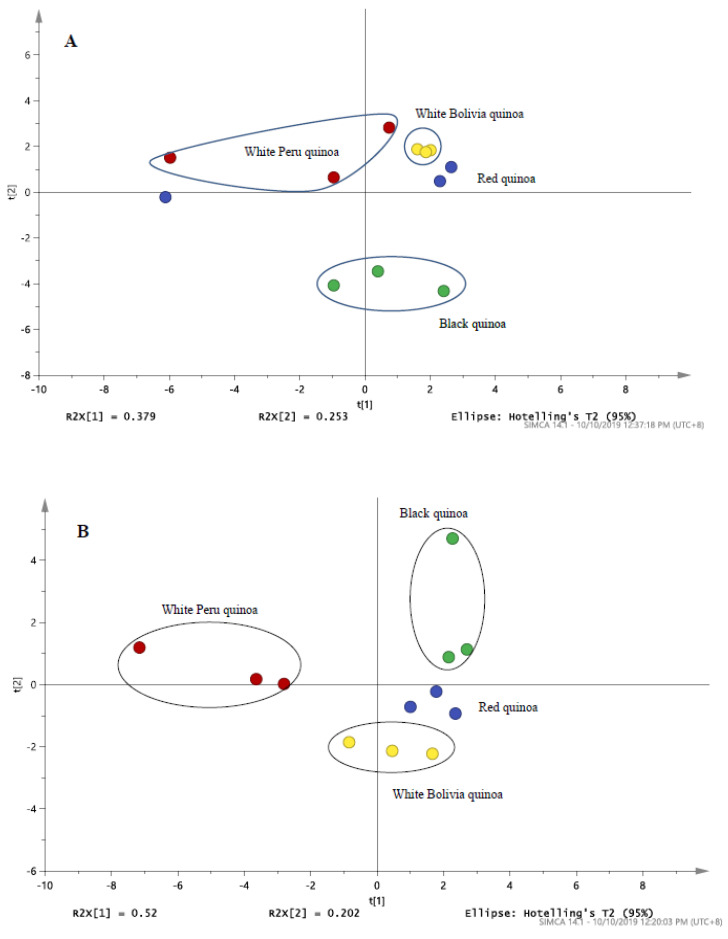
The PCA score plot of (**A**) LC/MS profile for black, red and white (Peru and Bolivia) quinoa derived from Table 1 (**B**) LC/UV profile for black, red and white quinoa detected at 280 nm.

**Figure 5:**
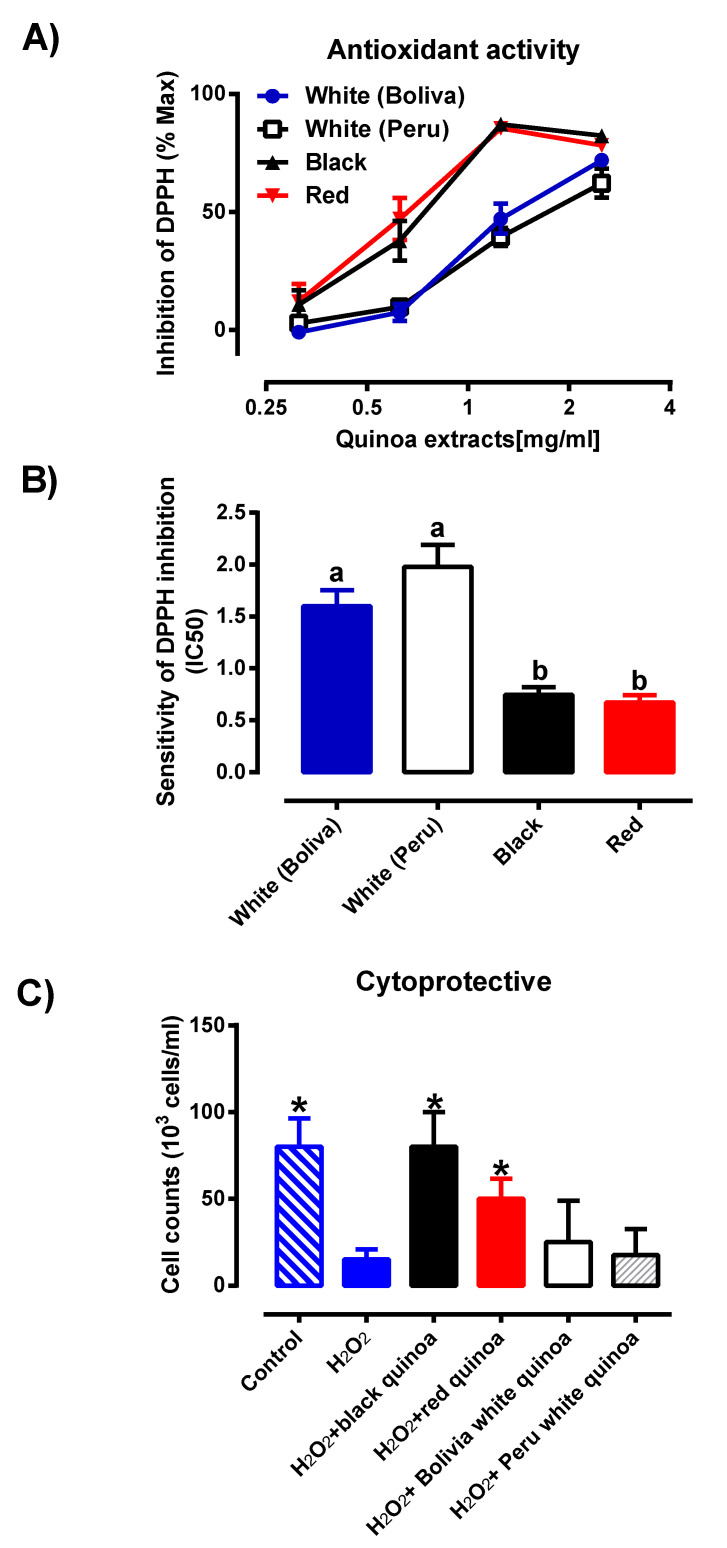
(**A**) Percentage of inhibition of pulverised quinoa seeds from the black, red and white pulverised seed extracts (Bolivia and Peru) were investigated (*n* = 6), where each result is represented by mean ± SD with the significance of *p* ≤ 0.05. (**B**) Sensitivity of DPPH assay of pulverised quinoa seeds from the black, red and white pulverised seed extracts (Bolivia and Peru) were investigated (*n* = 6), where each result is expressed as mean ± SD. Different letters (a, b) represents significantly different (*p* ≤ 0.05) from one another (**C**) Protective effects of extracts from quinoas on H_2_O_2_-induced cytotoxicity on the HMEC-1 cell line. HMEC-1 cells were administered with treatment medium over a period of 48 h. Cell viability was determined by cell counting (*n* = 4) before expressing as a cell concentration (cells/mL). Each value represents mean ± SD with the significance accepted at *p* ≤ 0.05. * Significantly different between cells treated with H_2_O_2_ alone (one-way ANOVA, Tukey’s test).

Based on the PCA score plot from LC/MS and LC/UV in Figure 4, the results of the pulverised quinoa variety portrayed a parallel comparison. With reference to Figure 3 and Figure 4, it was noted that higher variability was observed for the LC/MS data as compared to LC/UV. Despite the differences in seed color, the proximity of white Bolivian and red quinoa pinpoints a degree of congruency in the phytochemical profiles. Meanwhile, black and white Peruvian seeds were largely distinct from each other and the latter. The results indicated that by grinding the quinoas, little variation in antioxidant compounds within the same species of quinoa is experienced, reinforcing the concept that grounded quinoa ensures better result reproducibility than seeds. In Figure 5, the concentration response of these quinoa varieties of black, red and white (Peru and Bolivia) were also investigated for the DPPH inhibition activity. All of the quinoa varieties produce concentration-dependent inhibition of DPPH, indicating the antioxidant activity of the quinoa extracts (Figure 5A). The concentration response curve of the red and black quinoa was shifted to the left, which is indicative that the black and red quinoa had the highest radical inhibition activity in comparison to the white quinoa (Bolivia and Peru). Similarly, the IC_50_ values of the concentration response curves were calculated and shown in Figure 5B. The black and red quinoa extracts have comparable IC_50_ values, which were significantly lower than the white quinoa (Bolivia and Peru). To further characterize the antioxidant effects of black, red and white quinoa extracts, another in vitro ABTS antioxidant capacity assay was used. Based on the ABTS antioxidant capacity assay, the CEAC of the black, red and white quinoa was found to be 355.5 ± 31.9 µM (*n* = 3), be 284.2 ± 10.6 µM (*n* = 3) and 131.3 ± 13.0 µM (*n* = 3), respectively. Consistent with the DPPH antioxidant assay, the CEAC of the black and red quinoa were significantly higher (*p* < 0.05, 1-way ANOVA, Tukey’s test) than the white quinoa. Therefore, both in vitro antioxidant assays suggest that the black and red quinoa have a higher antioxidant capacity as compared to white quinoa.

Previous studies have relied on several different techniques to extract bioactive chemicals from quinoa seeds [3,4]. PHWE is increasingly recognized as a green and sustainable method for the extraction of botanicals [18,19], which is currently used in this study. Therefore, the extractable chemicals compounds obtained from PHWE may be different other extraction techniques used. For example, one of an important class of compounds, anthocyanins are widely reported to be a potent antioxidant and possess other biological effects, which could be found in the colored quinoa seeds [1,5]. Unfortunately, in this study, based on the amount of sample used, anthocyanins in all the variety of quinoa seeds at 100 °C were not detected. Hence, it remains inconclusive if anthocyanins can be extracted by using PHWE, and perhaps further optimization of extraction conditions for anthocyanins will be required in future studies. In the current study, the distribution of free (soluble) polyphenols and bounded polyphenols was not evaluated, as the current work focused on what is extractable from different types of quinoa. This could be another important limitation of this study, as bounded polyphenol (such as phenolic acid derivatives) is more important from a nutritional point of view. Despite the limitations, our study utilized the PHWE method for the extraction of quinoa seeds, which has detected the presence of the list of phytochemical compounds (Tables S1 and S2). More importantly, the quinoa extracts possessed antioxidant activities which are different between quinoa varieties.

### 3.4. Cytoprotective Effects of Plant Extracts against HMEC-1 Cell Line

In order to assess if the antioxidant activity of the quinoa extracts was able to protect against oxidative stress-induced cytotoxicity, HMEC-1 cells were treated with H_2_O_2_ and co-incubated with the variety of quinoa extracts. From Figure 5C, upon treatment with H_2_O_2,_ the cell concentration was significantly reduced (*p* < 0.05) when compared to the untreated control, indicating that H_2_O_2,_-induced cellular toxicity. Furthermore, the co-treatment of cells with either the black quinoa or red quinoa extract significantly increased (*p* < 0.05) cell concentration in comparison to H_2_O_2_ alone, indicating that both the black quinoa and red quinoa extract were able to prevent oxidative stress-induced cytotoxicity, which is likely attributable to their antioxidant capacity. Despite their antioxidant capacity, albeit weaker, the co-treatment of the white quinoa (Bolivia and Peru) caused a modest increase in cell concentration, which failed to reach statistical significance. Therefore, it was proposed that the distinctive profile for the black or red quinoa obtained in Figure 4 corresponded closely with the in vitro antioxidant assays (DPPH and ABTS) and cytoprotective effect (Figure 5C).

Phytochemicals with anti-oxidative nature are known non-essential nutrients with diverse health benefits which can curb the effects of oxidative stress [15]. Among the latter, black quinoa emerged as the variety containing the highest antioxidant capacity in the DPPH and ABTS assay. There have been reports that dark-coloured quinoa has a substantially higher concentration of antioxidants than lighter-coloured ones [4]. Although comparison with other methods of extraction were not performed, the data presented for the antioxidant and cytoprotective assay showed a similar trend as seen in other reports [3]. It was noted the antioxidant properties of the extracts produced may not be as comparable with that from organic solvent. However, DPPH data presented for the small amount of samples extracted from different types of quinoa were consistent with what was seen with the calibration range of ascorbic acid (5 to 30 µM) reported [39]. Finally, it was noted that the combination of chemical standardization with in vitro antioxidant and cytoprotective assay provided an effective approach for the evaluation of botanical extracts as compared to the analysis of chemical compounds from different methods of extraction [17].

Though the DPPH and ABTS experiments are a convenient assay in determining antioxidant activity, they can only provide an overall idea and cannot be used as a reliable tool due to the lack of in vitro conditions like pH and temperature, bioavailability, uptake and metabolism of antioxidant components [5]. As such, the cell viability assay was introduced, utilizing H_2_O_2_ to stimulate an oxidative stress environment. Though H_2_O_2_ is a non-radical, administration to a living organism can easily develop free radical reactions which are used as a precursor to producing reactive oxygen species that can cross cell membranes to react with Fe^2+^ and Cu^2+^ to form a cytotoxic hydroxyl-free radical [16,40]. Hence, the cytoprotective assay using H_2_O_2_ is, at best, an indirect assessment of the antioxidant capacity of the extracts in a cellular context. The limitation of this study would be the lack of direct measurement of the antioxidant capacity of the quinoa extracts in the cells, which could be more included in future studies on disease models, where there are elevated levels of oxidative stress. Despite the limitation and within the context of our experimental conditions, our study reported that the black or red quinoa extract exhibited the strongest antioxidant activity and complemented the observation from the cytoprotective assay in the cellular system.

## 4. Conclusions

The current work revealed that the types of phytochemicals and characteristics of different quinoa extracts were found to contribute to the antioxidant activity. PHWE provided an organic solvent-free approach for the production of quinoa extracts. Chemical fingerprint using separation tools with pattern recognition tools provided an alternative for the chemical standardization of botanical extracts. Instead of comparing the extraction efficiency with classical methods of extraction, the combination of chemical standardizations such as LC/UV and LC/MS, and pattern recognition tools such as PCA and biological assay, provided an approach for the evaluation and production of botanical extracts for functional food.

## Figures and Tables

**Table 1:** Normalised peak area of the pulverised, black, red and white (Bolivia and Peru) derived from LC/MS analysis. Data expressed as mean ± SD (*n* = 3).

Name of Compound	[M-H]-	MSMS	Normalised Peak Area			
	*m*/*z*	*m*/*z*	Black	Red	White (Peru)	White (Bolivia)
Vanillic acid	167	151, 125, 106, 80, 58	0.00695 ± 0.00245	0.0085 ± 0.00251	0.00152 ± 0.000946	0.00468 ± 0.000122
Vanillic acid glucoside	329	151, 133, 109	0.0108 ± 0.00292	0.0488 ± 0.0254	0.0179 ± 0.00829	0.0726 ± 0.00478
Vanillin	151	108, 80, 66, 61	0.0173 ± 0.00561	0.0165 ± 0.0144	0.235 ± 0.317	0.0547 ± 0.00677
p-coumaric acid	163	99, 91, 73, 71, 69, 57	0.102 ± 0.0272	0.0272 ± 0.0136	0.0166 ± 0.00801	0.0482 ± 0.00109
Ferulic acid	193	147, 128, 113, 89, 77,75, 71, 67, 59, 57, 55	0.0162 ± 0.00252	0.0145 ± 0.00840	0.0399 ± 0.00928	0.0288 ± 0.00197
Caffeic acid	179	89, 71, 59, 57	0.285 ± 0.0592	0.268 ± 0.0309	0.267 ± 0.125	0.230 ± 0.0175
Catechin	289	171, 157, 145, 133,127, 111, 106	0.0667 ± 0.0178	0.0318 ± 0.0166	0.0354 ± 0.0153	0.104 ± 0.00879
Daidzein	263	230, 166, 146, 124, 110, 107, 102	0.00564 ± 0.00183	0.00167 ± 0.000792	0.00145 ± 0.000543	0.0022 ± 5.8 E-05
Genistein	269	197, 173, 157, 143, 139, 121	0.0106 ± 0.00223	0.00768 ± 0.00396	0.00835 ± 0.00299	0.0137 ± 0.00157
Quercetin	301	193, 168, 150, 125, 107	0.00436 ± 0.00152	0.00448 ± 0.00244	0.00290 ± 0.00135	0.005 ± 0.000557
Quercetin 3-rutinoside	609	300, 285, 287, 257, 137, 114	0.0348 ± 0.00984	0.0570 ± 0.0301	0.0041 ± 0.00156	0.0528 ± 0.013
Unknown	431		0.185 ± 0.0174	0.135 ± 0.0736	0.142 ± 0.0701	0.157 ± 0.00459
Unknown	319	242, 206, 189, 160, 133, 125	0.0216 ± 0.00346	0.0205 ± 0.0105	0.0151 ± 0.00692	0.0304 ± 0.00331
Unknown	726	726, 284	0.00663 ± 0.00140	0.00483 ± 0.0006	0.0551 ± 0.0241	0.0288 ± 0.00412
Unkownn	479	389, 318, 258, 139	0.00226 ± 0.000549	0.00613 ± 0.00342	0.00505 ± 0.00208	0.00732 ± 0.000860

## Supplementary Materials

The following are available online at https://www.mdpi.com/2076-3921/9/11/1110/s1, Table S1: Peak area of LC/MS analysis on 1.00 g of pulverised and non-pulverised white Bolivian seeds extracted at 80, 100 and 120 °C, Table S2: Normalised peak area of LC/MS analysis of pulverised and non-pulverised white Bolivian seeds based on the average peak area of the LC/MS data in Table S1, Figure S1: Instrumental setup for pressurized hot water extraction (PHWE) system, Figure S2: Normalized peak intensity by XRF from red (A), black (B), Bolivia white (C) and Peru white (D) quinoa, Figure S3: Comparison of normalized peak intensity of potassium (A) and Calcium (B) between red, black, Bolivia white and Peru white quinoa, Figure S4: LC-UV-MS chromatograms of black, red and white quinoas. A and B: Black quinoa, C and D: Red quinoa, E and F G: White Peruvian quinoa, G and H: White Bolivian quinoa. 1: LC-MS, 2: LC-UV detected at 280nm.
