# Supplementary material for: Antioxidant and Cytoprotective Effect of Quinoa (Chenopodium quinoa Willd.) with Pressurized Hot Water Extraction (PHWE)
Source: Antioxidants (Basel). 2020 Nov 11;9(11):1110. doi: 10.3390/antiox9111110 (PMC7697190; doi:10.3390/antiox9111110)
Supplement: Supplementary file 1 [file antioxidants-09-01110-s001.pdf]

Supplementary information

Table S1, Peak area of LC/MS analysis on 1.00 g of pulverised and non-pulverised white Bolivian seeds extracted at 80, 100 and 120°C.

Name of compound	[M-H]-	MSMS	PHWE at 80°C		PHWE at 100°C		PHWE at 120°C	
	m/z	m/z	Pulverised	Seed	Pulverised	Seed	Pulverised	Seed
Vanillic acid	167	151,125,106,80, 58	429285 ± 26560	7343251 ± 312529	423620 ± 83732	61083684 ± 71992679	502747 ± 118151	9304894 ± 996688
Vanillic acid glucoside	329	151,133, 109	23529169 ± 1232595	26198220 ± 3361363	23221184 ± 1989702	2887539 ± 1546413	24488878 ± 2850139	4808947 ± 3752363
Vanillin	151	108,80,66,61	24054806 ± 2876132	14701647 ± 3895764	17496702 ± 2445193	13583596 ± 952963	11550842 ± 3163558	17884142 ± 3536010
p-coumaric acid	163	99,91,73,71,69,57	15525574 ± 891590	2816675 ± 401223	15419089 ± 615817	2384406 ± 255870	16011481 ± 1485634	2454211 ± 298439
Ferulic acid	193	147, 128, 113, 89, 77,75, 71, 67,59,57,55	6909818 ± 1547141	3321839 ± 1268691	9175112 ± 412082	3204020 ± 96495	12243099 ± 5204634	3316394 ± 543556
Caffeic acid	179	89, 71, 59, 57	71004668 ± 7488133	2006711 ± 188797	73292001 ± 3996372	2268681 ± 98687	97066891 ± 27878225	1993441 ± 44515
Catechin	289	171, 157, 145, 133,127, 111, 106	39767749 ± 2120859	855751 ± 97457	33305070 ± 3521007	1134720 ± 192802	32103345 ± 2451508	1310063 ± 375367
Daidzein	263	230, 166, 146, 124, 110, 107, 102	754375 ± 75626	1272921 ± 151480	703069 ± 28294	1787250 ± 183048	776080 ± 167326	1312328 ± 83216
Genistein	269	197, 173, 157, 143, 139, 121	4301403 ± 223513	1110627 ± 91027	4354205 ± 386528	1138212 ± 171189	4515149 ± 764320	1087311 ± 16170
Quercetin	301	193, 168,150, 125, 107	1448989 ± 65609	1657788 ± 649599	1593519 ± 136412	1448169 ± 140901	1874591 ± 196472	1854812 ± 914458
Quercetin 3-rutinoside	609	300, 285, 287, 257, 137, 114	17623074 ± 3972165	724508 ± 336429	16960368 ± 4479701	575094 ± 41257	14262067 ± 2046964	821165 ± 110140
Unknown	431		47572042 ± 3440460	3703383 ± 796058	50068554 ± 262786	4238304 ± 96782	47773038 ± 6900434	4952239 ± 337837
Unknown	319	242, 206, 189, 160, 133, 125	9404690 ± 859827	2257689 ± 389281	9687765 ± 836753	2168055 ± 261909	8251738 ± 196762	2227992 ± 105674
Unknown	726	726,284	10305678 ± 1256805	992904 ± 452820	9245759 ± 1496059	765332 ± 39072	9626085 ± 561538	1462503 ± 398794
Unkownn	479	389, 318, 258, 139	2265209 ± 95452	708803 ± 288759	2332930 ± 215038	623832 ± 52634	2452477 ± 398245	821918 ± 20530

Results were expressed as a mean of a triplicate measurement ± SD (n=3).

Table S2, Normalised peak area of LC/MS analysis of pulverised and non-pulverised white Bolivian seeds based on the average peak area of the LC/MS data in Table S1.

Name of compound	[M-H]- m/z	MSMS m/z	PHWE at 80°C		PHWE at 100°C		PHWE at 120°C	
			Pulverised	Seed	Pulverised	Seed	Pulverised	Seed
Vanillic acid	167	151,125,106,80, 58	0.00119 ± 9.94E-05	0.0918 ± 0.0153	0.00117 ± 0.000203	0.363 ± 0.278	0.00119 ± 0.000459	0.141 ± 0.0324
Vanillic acid glucoside	329	151,133, 109	0.0651 ± 0.00146	0.0323 ± 0.0392	0.0642 ± 0.00576	0.0396 ± 0.0319	0.0557 ± 0.0146	0.0643 ± 0.0411
Vanillin	151	108,80,66,61	0.0668 ± 0.0086	0.176 ± 0.011	0.0482 ± 0.00541	0.169 ± 0.0767	0.0276 ± 0.0114	0.260 ± 0.0141
p-coumaric acid	163	99,91,73,71,69,57	0.0430 ± 0.00118	0.0345 ± 0.0019	0.0426 ± 0.000879	0.0303 ± 0.0147	0.0367 ± 0.0101	0.0360 ± 0.001
Ferulic acid	193	147, 128, 113, 89, 77,75, 71, 67,59,57,55	0.0191 ± 0.00386	0.0389 ± 0.00688	0.0254 ± 0.0017	0.0404 ± 0.0185	0.0278 ± 0.014	0.0487 ± 0.00565
Caffeic acid	179	89, 71, 59, 57	0.196 ± 0.0158	0.0248 ± 0.0031	0.203 ± 0.0157	0.0278 ± 0.118	0.225 ± 0.0875	0.0298 ± 0.00423
Catechin	289	171, 157, 145, 133,127, 111, 106	0.110 ± 0.00813	0.0108 ± 0.00256	0.0919 ± 0.00768	0.0150 ± 0.00786	0.0756 ± 0.0243	0.0200 ± 0.0078
Daidzein	263	230, 166, 146, 124, 110, 107, 102	0.00209 ± 0.000221	0.0158 ±0.00276	0.00194 ± 0.000101	0.0213 ± 0.00851	0.00169 ± 0.000195	0.0195 ± 0.0027
Genistein	269	197, 173, 157, 143, 139, 121	0.0119 ± 0.000271	0.0138 ± 0.00269	0.0121 ± 0.00124	0.0132 ± 0.00484	0.0126 ± 0.00114	0.0162 ± 0.00201
Quercetin	301	193, 168,150, 125, 107	0.00402 ± 0.000284	0.0196 ± 0.00436	0.00441 ± 0.000447	0.0175 ± 0.00742	0.00428 ± 0.00106	0.0259 ± 0.00877
Quercetin 3-rutinoside	609	300, 285, 287, 257, 137, 114	0.0488 ± 0.0108	0.00802 ± 0.00209	0.0468± 0.0121	0.00940 ± 0.000575	0.0337 ± 0.0115	0.0121 ± 0.00106
Unknown	431		0.132 ± 0.0108	0.0449 ± 0.00382	0.138± 0.00352	0.0531 ± 0.0239	0.109 ± 0.0319	0.0732 ± 0.00558
Unknown	319	242, 206, 189, 160, 133, 125	0.0261 ± 0.00226	0.0279 ± 0.0052	0.0268 ± 0.00238	0.026 ± 0.0109	0.0193 ± 0.00611	0.033± 0.00318
Unknown	726	726,284	0.0285 ± 0.0031	0.0115 ± 0.003	0.0256 ± 0.00419	0.00932 ± 0.0039	0.0228 ± 0.00789	0.0210 ± 0.0025
Unkownn	479	389, 318, 258, 139	0.00628 ± 0.000324	0.00746± 0.00204	0.00646 ± 0.000708	0.00629 ± 0.00315	0.00586± 0.00249	0.0122± 0.00142

Results were based on the mean of a triplicate measurement ± SD (n=3).

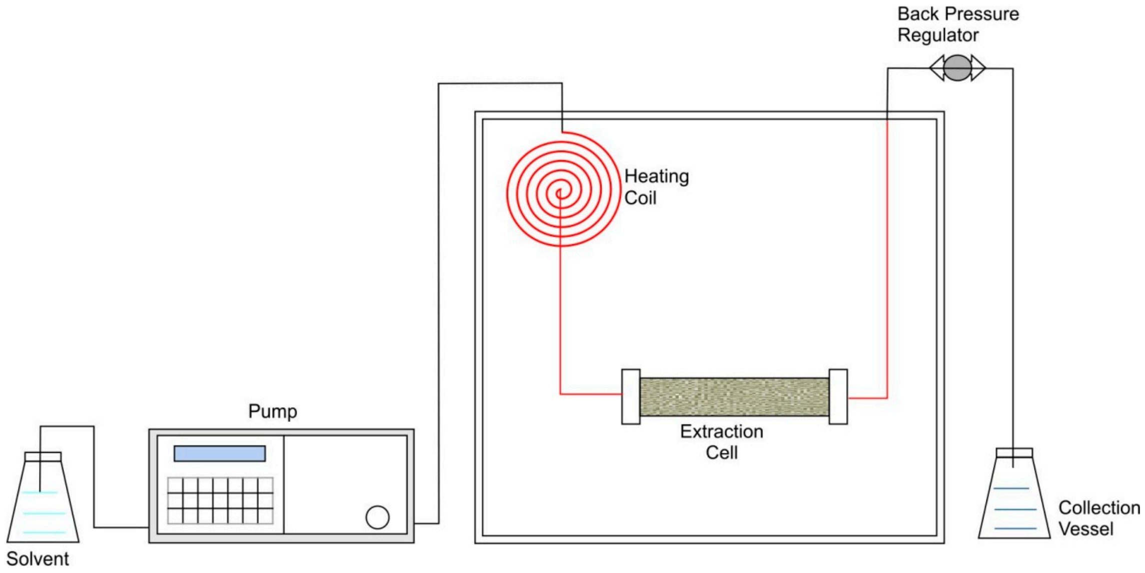

Figure S1. Instrumental setup for pressurized hot water extraction (PHWE) system.

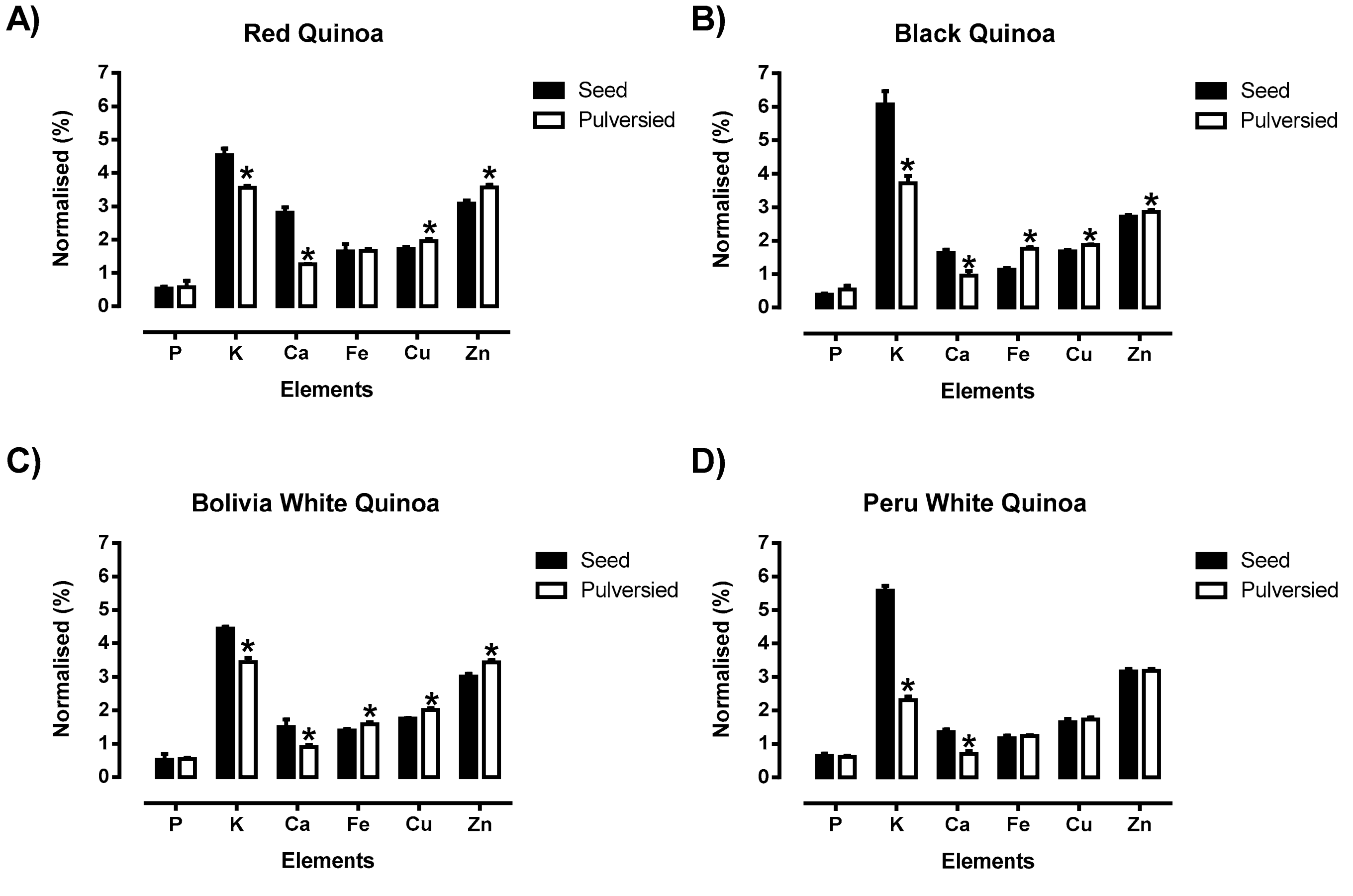

Figure S2: Normalized peak intensity by XRF from red (A), black (B), Bolivia white (C) and Peru white (D) quinoa. * Significant difference ($p \leq 0.05$) between whole seed and pulverized seeds from black, red and white quinoa. Pulverization of the quinoa significantly decreased K and Ca when compared to whole seed. However, in most cases with the exception of white quinoa (Peru), other elements such as Fe, Cu and Zn were significantly increased in the pulverized seeds when compared to whole seeds. Data are expressed as mean ± SD (n=3).

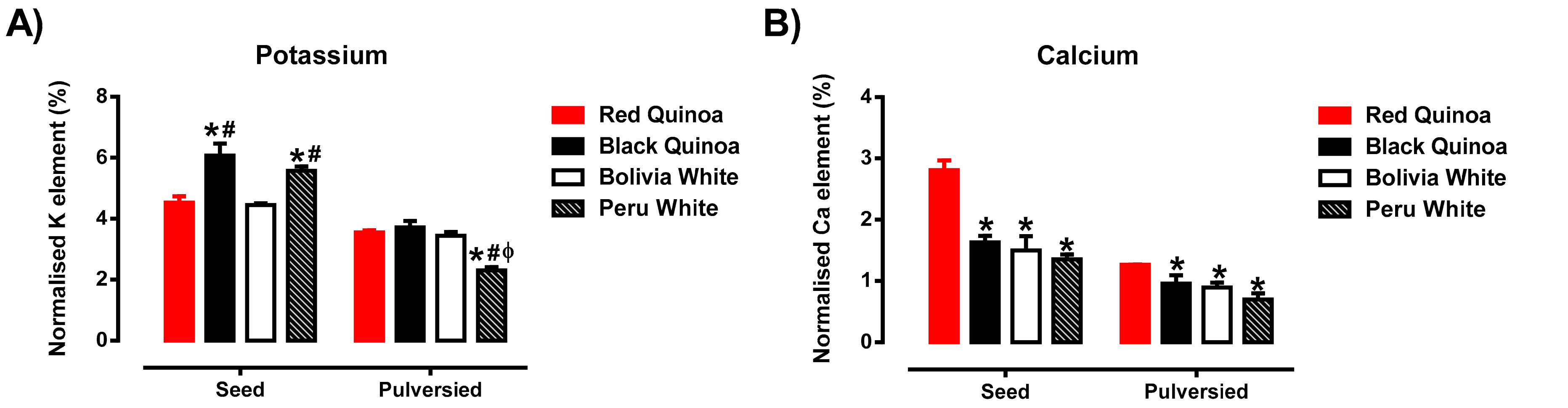

Figure S3: Comparison of normalized peak intensity of potassium (A) and Calcium (B) between red, black, Bolivia white and Peru white quinoa. * Significant difference ($p \leq 0.05$) between red quinoa within whole seed and pulverized seeds. # Significant difference ($p \leq 0.05$) between Bolivia white quinoa within whole seed and pulverized seeds. Φ Significant difference ($p \leq 0.05$) between black quinoa within whole seed and pulverized seeds. Data are expressed as mean ± SD (n=3).

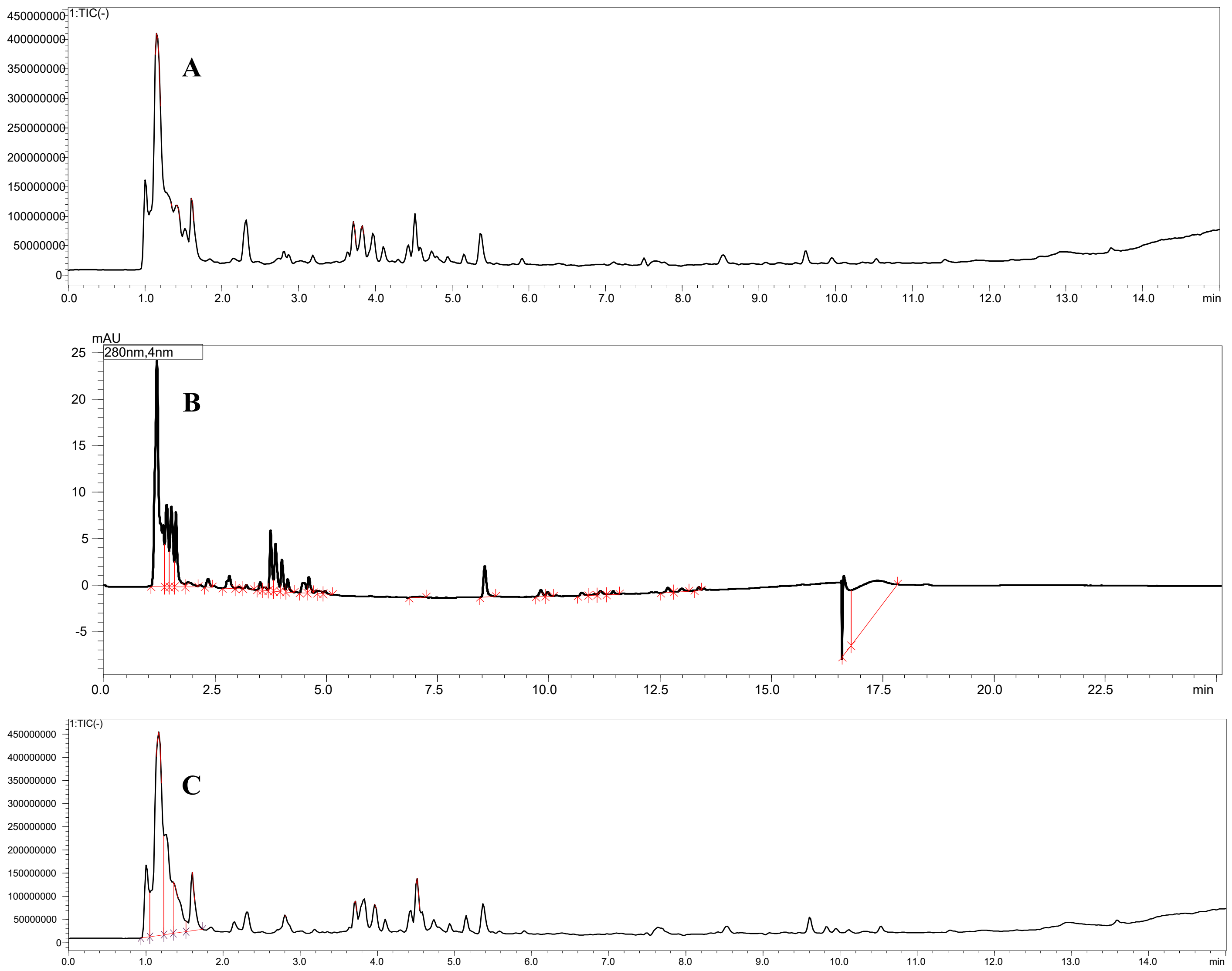
A
1:TIC(-)
450000000
400000000
350000000
300000000
250000000
200000000
150000000
100000000
50000000
0
0.0 1.0 2.0 3.0 4.0 5.0 6.0 7.0 8.0 9.0 10.0 11.0 12.0 13.0 14.0 min
B
mAU
280nm,4nm
25 20 15 10 5 0 -5
0.0 2.5 5.0 7.5 10.0 12.5 15.0 17.5 20.0 22.5 min
C
1:TIC(-)
450000000
400000000
350000000
300000000
250000000
200000000
150000000
100000000
50000000
0
0.0 1.0 2.0 3.0 4.0 5.0 6.0 7.0 8.0 9.0 10.0 11.0 12.0 13.0 14.0 min

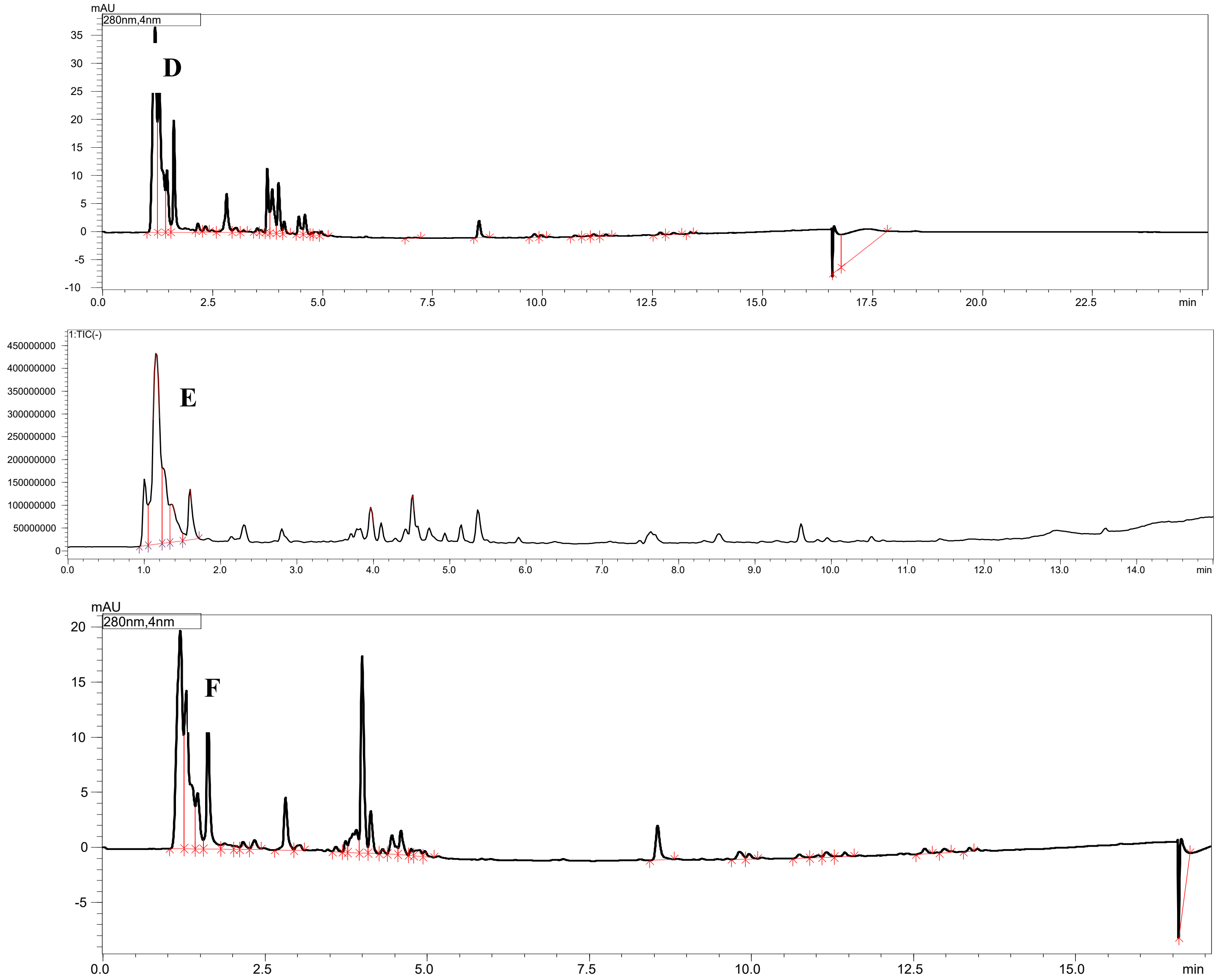

mAU
280nm,4nm
D
35
30
25
20
15
10
5
0
-5
-10
0.0
2.5
5.0
7.5
10.0
12.5
15.0
17.5
20.0
22.5
min
1:TIC(-)
E
450000000
400000000
350000000
300000000
250000000
200000000
150000000
100000000
50000000
0
0.0
1.0
2.0
3.0
4.0
5.0
6.0
7.0
8.0
9.0
10.0
11.0
12.0
13.0
14.0
min
mAU
280nm,4nm
F
20
15
10
5
0
-5
0.0
2.5
5.0
7.5
10.0
12.5
15.0
min

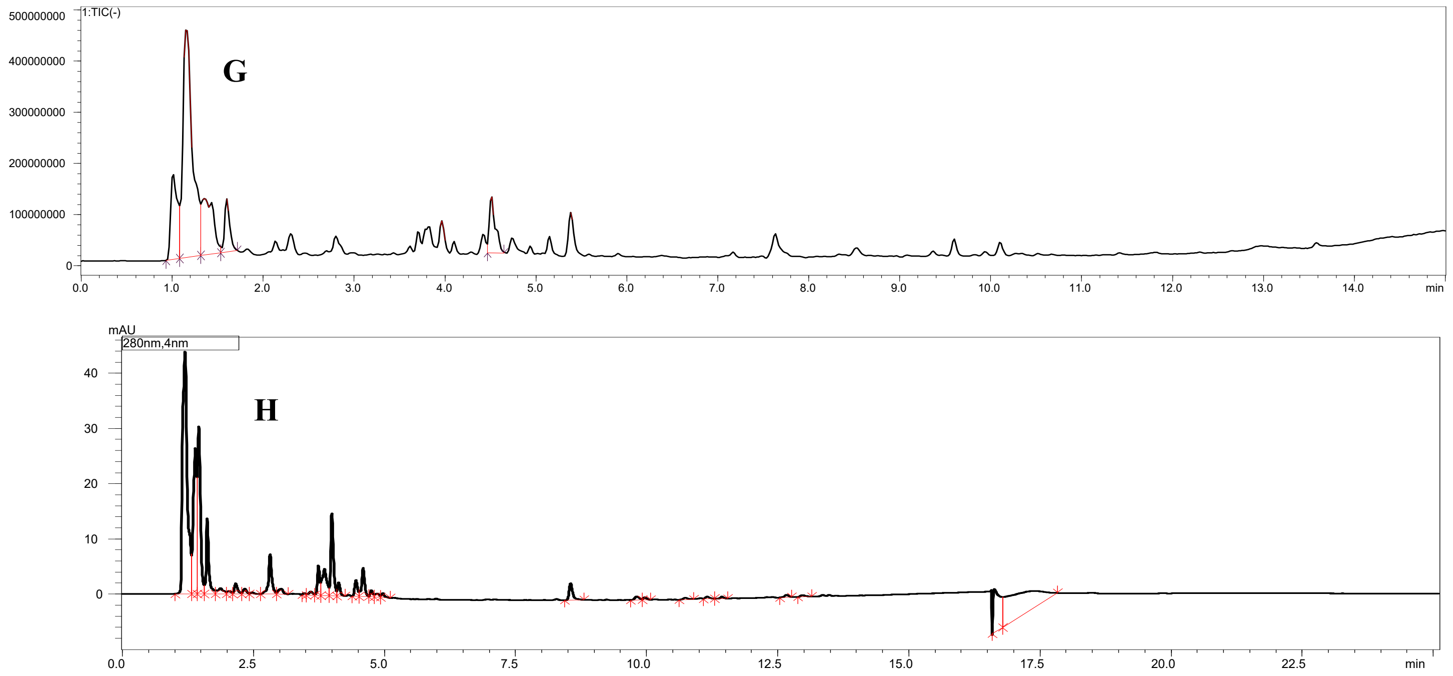

Figure S4. LC-UV-MS chromatograms of black, red and white quinoas. A and B: Black quinoa, C and D: Red quinoa, E and F G: White Peruvian quinoa, G and H: White Bolivian quinoa. 1: LC-MS, 2: LC-UV detected at 280nm.
